# Effort-reward imbalance and its association with health among permanent and fixed-term workers

**DOI:** 10.1186/1751-0759-4-16

**Published:** 2010-11-05

**Authors:** Mariko Inoue, Shinobu Tsurugano, Mariko Nishikitani, Eiji Yano

**Affiliations:** 1 Department of Hygiene and Public Health, School of Medicine, Teikyo University 2-11-1 Kaga, Itabashi-ku, Tokyo, 173-8605, Japan

## Abstract

**Background:**

In the past decade, the changing labor market seems to have rejected the traditional standards employment and has begun to support a variety of non-standard forms of work in their place. The purpose of our study was to compare the degree of job stress, sources of job stress, and association of high job stress with health among permanent and fixed-term workers.

**Methods:**

Our study subjects were 709 male workers aged 30 to 49 years in a suburb of Tokyo, Japan. In 2008, we conducted a cross-sectional study to compare job stress using an effort-reward imbalance (ERI) model questionnaire. Lifestyles, subjective symptoms, and body mass index were also observed from the 2008 health check-up data.

**Results:**

The rate of job stress of the high-risk group measured by ERI questionnaire was not different between permanent and fixed-term workers. However, the content of the ERI components differed. Permanent workers were distressed more by effort, overwork, or job demand, while fixed-term workers were distressed more by their job insecurity. Moreover, higher ERI was associated with existence of subjective symptoms (OR = 2.07, 95% CI: 1.42-3.03) and obesity (OR = 2.84, 95% CI:1.78-4.53) in fixed-term workers while this tendency was not found in permanent workers.

**Conclusions:**

Our study showed that workers with different employment types, permanent and fixed-term, have dissimilar sources of job stress even though their degree of job stress seems to be the same. High ERI was associated with existing subjective symptoms and obesity in fixed-term workers. Therefore, understanding different sources of job stress and their association with health among permanent and fixed-term workers should be considered to prevent further health problems.

## Background

In the past decade, the changing labor market seems to have diminished the traditional standards of employment and a variety of non-standard forms of work have emerged in their place. To overcome recession and a high unemployment rate, national labor market policy began to support work sharing, deregulation of employment, and flexibility of work in developed countries [1]. These non-standard forms of work are known as precarious employment (or contingent work, firstly introduced by Freedman [2]). World Health Organization Commission on Social Determinants of Health, Employment Conditions Knowledge Network, described precarious employment as "the lacking of the relations that support the standard employment relationship, making workers more vulnerable in jobs that are unstable, unprotected and increasingly unable to sustain individuals and families." [3]. This commission also defined four characteristics of precarious employment in terms of high job insecurity, low wage level, lack or limited social benefits, and powerlessness. Quinlan et al. selected five categories of precarious employment that include temporary workers with short-term contracts, workers who experienced organizational change (e.g., downsizing, restructuring, privatization, etc.), outsourcing or home-based workers, part-time workers, and small business workers [4]. Workers with precarious employment (precarious workers) are not always considered inferior when supported by welfare and labor support system [5]; however, it is also true that precarious employment is counted as one of the major social determinants of health disrupting decent work [6].

Japan is one of the countries that increased the use of precarious workers. It must be surprising that the Japanese employment system, which values workers' lifetime commitment to work (especially working for only one company), is not as common as it was decades ago. The current Japanese statistics define precarious workers as non-regular workers, including part-time workers, temporary workers, dispatched workers from temporary labor agency, contractors/entrusted employees, and other related workers. Among the workforce in Japan, with the exception of managers and self-employed people, 33.7% were categorized as precarious workers in 2009 compared to 20.2% in 1990 [7]. This increase was based on the deregulation of non-regular employees and lack of regular employment opportunities for new graduates during the recession period in late 1990s and 2000s. Precarious workers were employed as a convenient labor force, which means that the number of employees can be adjusted depending on the job demand in the company, which is quite unstable. This situation is currently indeed threatening Japanese workers' mental health [8].

Several researchers have found that precarious employment was associated with vulnerable mental health. A meta-analysis concluded that mental morbidity increased more among temporary workers compared to permanent workers [9]. Cohort studies on the mental health among permanent and precarious workers measured by General Health Questionaire-12 (GHQ-12) concluded that people who obtained permanent employment from precarious work experienced less psychological distress [10]. Other cross-sectional studies using GHQ-12 showed that temporary manual male workers had poorer mental health compared to permanent male workers [11]. Considering other aspects of mental health, part-time workers were more likely to engage in suicidal ideation in Canada [12]. Finnish study has analyzed differences in treatment seeking behavior by employment status and reported that temporary workers received more prescriptions for antidepressant medicines compared to permanent workers [13]. According to International Classification of Diseases (ICD-10), female precarious workers had more psychiatric morbidity [14]. Center for Epidemiologic Studies Depression Scale (CES-D) reported that non-preferable part-time workers were more likely to experience depression compared to workers without any part-time work experience [15]. On the contrary, several studies reported opposite findings, indicating that fixed-term workers had lower psychological distress as measured by GHQ-12 [16]. Some studies reported that permanent workers experience greater stress compared to precarious workers [17,18]. Morbidity of minor psychiatric disorders did not differ for permanent and precarious workers [19].

In Japan, a limited number of studies have examined and compared precarious workers' health to permanent workers, and their findings were inconclusive. One study suggested that regular employees reported more job pressures [20] while the prevalence of major depression among fixed-term and permanent workers did not differ [21]. Another study showed that male part-time workers and female temporary or contract workers experienced greater psychological distress [22].

Validated and standardized measures were used in several studies to determine the mental health of precarious workers; however, the studies did not distinguish the sources of stress, that is, occupational and personal sources. The effect on the mental health of precarious workers from job strain was measured as one aspect of occupational stress [23]. Another aspect of job stress, effort-reward imbalance (ERI), and its association with health by employment status has not been discussed; although, the use of the ERI questionnaire has been recommended as appropriate for comparing permanent and precarious workers on work related distress [24]. Therefore, the first purpose of our study was to compare job stress and to investigate the sources of stress between permanent and precarious workers. The second purpose was to investigate whether high ERI relates to subjective symptoms and obesity as the causes of further health concerns.

## Methods

We conducted a cross-sectional study to compare different types of workers job stress, using the effort-reward imbalance model (ERI) questionnaire, and health status in 2008. The study design was approved by Teikyo University School of Medicine Review Board.

### Study Population

Study subjects were employees at a research institute in a suburb in Tokyo working as either clerks or researchers with permanent or fixed-term with limited term employment contracts. The fixed-term work is considered precarious employment. There were 1,884 workers (529 permanent workers and 1,355 fixed-term workers) in 2008. We obtained data from the annual health examination conducted in 2008. Overall, 1,706 individuals took part in the health examination. We focused on the middle-aged population, 30-49 years old (n = 1,004), because the majority of workers in their twenties did not have tenure while the permanent or fixed-term employment status was clearly defined among workers in the middle stage of their career. Based on these inclusion criteria, female workers (n = 295) represented the minority and could not be examined in further analysis (264 were fixed-term workers and 31 were permanent workers); therefore, they were excluded from our study. The data of 709 male workers were used in this study.

### Health examination

Under the Industrial Safety and Health Act, it is mandatory for all employees in Japan to have an annual health examination. From the results of the health examination, we obtained information about lifestyle, subjective symptoms, height, and weight to calculate body mass index (BMI) of the subjects.

Regarding lifestyles, we obtained the data for smoking, alcohol intake frequency, physical exercise, sleeping hours, and working hours. The questionnaire categorized smoking status as smoker, ex-smoker, or non-smoker. For the purpose of our analysis, we categorized subjects into two groups, either as a current smoker or as a non-smoker. Those who drank alcohol more than four days per week were categorized as drinkers. Those who engaged in exercise more than twice a week were categorized as having a habit of physical exercise. Time spent sleeping was grouped into five levels (<4, 4-5, 5-6, 6-7, >7 hours). People who were grouped into the 'longer time spent sleeping' category slept for >6 hours. Working hours were calculated from self-reported usual starting and ending time of work.

Subjective symptoms were queried by asking whether the subject had any symptoms within past month. An example including 31 symptoms was shown in the questionnaire. They include headache, dizziness, stomachache, cough, backache, and related symptoms. Obesity was defined as BMI ≥25.0 kg/m^2^, according to the definitions of the Japan Society for the Study of Obesity [25]. BMI was calculated by measured height and weight.

### ERI model

The Japanese version of the ERI Questionnaire, an indirect indicator of occupational unfairness, measured job stress [26]. ERI and its effect on psychosomatic symptoms, cardiovascular diseases, and health related lifestyles were also investigated [27]. The ERI questionnaire contains 23-items measuring work related stress. Siegrist originally developed ERI to measure whether work effort corresponded with reward [28]. Each question on the ERI is measured on a five-point Likert scale assessing the degree of distress about each statement. The effort scale of the ERI consists of six items. A higher score indicates that greater perceived demands become stressful. Reward consists of three subcategories; namely, esteem reward, reward related to job security, and reward related to job promotion or financial incentives. Eleven items measure the reward. A higher score indicates greater perceived reward. The effort-reward ratio was calculated as a continuous variable. It can also be assessed on a dichotomous scale, with an effort-reward ratio ≥1 indicating a high-risk group and an effort-reward ratio < 1 indicating a low-risk group. It is recommended that the ERI score be compared by the use of this dichotomous variable. A standardized questionnaire should be used to assess the total score. However, it is true that each component of the ERI questionnaire includes valuable information to distinguish the sources of job stress, as seen in previous studies [29,30]. Therefore, we also compared scores for each component and subcategory of ERI questionnaire.

### Statistical analysis

The total ERI score and scores of effort, reward, and subcategories of reward (esteem, job promotion, and job security) for permanent and fixed-term workers were compared using the Wilcoxon rank-sum tests. High effort-reward ratios and lifestyles were compared using chi-square tests. Logistic regression analysis was performed to examine associations between higher ERI and health, existence of subjective symptoms, and obesity. The analysis was adjusted for age, occupational category, working hours, smoking, alcohol consumption, exercise, and sleeping hours. The ERI score was further divided into two to indicate lower and higher levels of job stress. This division was based on whether the ERI score was higher or lower than the median. The lower ERI group was used as a reference group in the logistic regression analysis.

## Results

Among 709 study subjects, 218 (30.7%) were permanent workers and 491 (69.3%) were fixed-term workers. Table 1 shows the basic characteristics of the study subjects. Permanent and fixed-term workers did not differ on working hours and health-related lifestyles while mean age between permanent and fixed-term workers differed. Since this study was conducted in a research institute, the majority of workers were researchers. Regarding health indicators, the proportion of workers who have subjective symptoms and who are obese was higher among permanent workers compared to fixed-term workers.

**Table 1:** Basic characteristics of study subjects

	permanent		fixed-term		*p *value
	(n = 218)		(n = 491)		
Age mean (SD)	41.3	(4.7)	35.8	(4.7)	<0.01
Occupation n(%)					
research	151	(69.3)	452	(92.1)	<0.01
clerk	67	(30.7)	39	(7.9)	
Working hours					
hours mean (SD)	11.3	(1.6)	11.3	(1.8)	0.74
Lifestyles n(%)					
smoking	34	(15.6)	79	(16.1)	0.87
alcohol	70	(32.1)	126	(25.7)	0.08
exercise	48	(22.0)	141	(28.7)	0.06
sleeping hours ≥6 hours	109	(50.0)	271	(55.2)	0.20
Health status n (%)					
have any symptoms	110	(50.5)	205	(41.8)	0.03
obesity	73	(33.5)	112	(22.8)	<0.01

Abbreviations: SD, standard deviation

Table 2 summarizes the responses from the ERI questionnaire. The proportion placed in the ERI high-risk group was the same for permanent and fixed-term workers. A greater number of permanent workers were categorized into the ERI high-risk group compared to fixed-term workers, although the result was not statistically significant. The average effort-reward ratio was higher among permanent workers compared to fixed-term workers (*p *< 0.01). The total score of the effort component was higher among permanent workers (*p *< 0.01) compared to fixed-term workers while the total score of the reward component was not different across the two groups (*p *= 0.53).

**Table 2:** Differences of effort reward imbalance questionnaire scores between permanent and fixed-term male workers

	permanent		fixed-term		*p *value
	(n = 218)		(n = 491)		
ERI risk groups n (%) ^a)^					
effort/reward ≧1	18	(8.3)	33	(6.7)	0.47
ERI scores ^b)^	median	(25-75%)	median	(25-75%)	
effort/reward ratio	0.49	(0.37 - 0.69)	0.39	(0.28 - 0.56)	<0.01
effort	13	(10 - 17)	10	(8 - 13)	<0.01
reward	49	(42 - 52)	49	(43 - 53)	0.53
*subcategories of reward*					
esteem	23	(20 - 25)	25	(21 - 25)	<0.01
job promotion	17	(15 - 19)	17	(14 - 19)	0.53
job insecurity	9	(7 - 10)	8	(6 - 9)	<0.01

Abbreviations: ERI, effort reward imbanlance

Based on analyzing the subcategories of the reward component, we found that the esteem component was more likely to distress permanent workers. On the contrary, job insecurity more likely distressed fixed-term workers compared to permanent workers. The other subcategory, job promotion, did not differ among permanent and fixed-term workers.

As shown in Table 3, among fixed-term workers, the higher ERI group was significantly more likely to experience subjective symptoms (OR = 2.07, 95% confidence interval (CI):1.42-3.03) and obesity (OR = 2.84, 95% CI: 1.78-4.53) while this tendency was not found for permanent workers.

**Table 3:** Subjective symptoms and obesity associated with higher effort-reward imbalance for permanent and fixed-term workers.

	permanent		fixed-term	
	OR	95% CI	OR	95% CI
*Subjective symptoms*				
Lower ERI	1.00		1.00	
Higher ERI	0.87	(0.47 - 1.61)	2.07	(1.42 - 3.03)
*Obesity*				
Lower ERI	1.00		1.00	
Higher ERI	1.90	(0.96 - 3.75)	2.84	(1.78 - 4.53)

Logistic regression analysis was adjusted for age, occupational category, working hours, smoking, alcohol consumption, exercise, and sleep

## Discussion

Permanent and fixed-term workers who are in the middle stage of their career did not differ on high-risk of job stress as measured by the ERI questionnaire. However, through detailed comparison of each ERI component, we found that these two types of employees suffer from different sources of occupational stress. Too much effort, such as overwork and job demand, seemed to distress permanent workers more while job insecurity seemed to distress fixed-term workers more. The same results were found even when we performed stratified analysis among clerical workers and researchers.

The proportion of workers in the high-risk ERI ratio group (ERI ≥ 1) was the same among permanent and fixed-term workers. Therefore, the seriousness of effort-reward imbalance of both permanent and precarious workers did not differ at all and both employment types experienced hardship from job stress. What differed between permanent and fixed-term workers were the sources of job stress. A greater proportion of permanent workers was distressed by the effort component compared to fixed-term workers. This result proved that permanent workers suffered distress from job demand, workload, and too much responsibility. These permanent workers experienced slight downsizing in this institute. Demand for permanent workers should therefore increase. We focused on a mid-career population in our study. These workers might experience a dilemma working with employees in senior positions in addition to working with younger workers.

Regarding reward subcategories, permanent workers complained more about esteem compared to fixed-term workers. Permanent workers were more distressed from not getting the respect from their seniors and colleagues based on our detailed analysis of each statement (not shown in the tables). Self-esteem of permanent workers who might perceive themselves superior might be mismatched. Our study subjects were middle-aged and had a moderate career, and the self-confidence of permanent workers might influence their perception of self-esteem. Even for fixed-term workers, this institution seems to have a supportive organizational culture because fixed-term workers with higher self-esteem experienced less job stress. A possible reason is that workers in research or academic fields usually start their job as precarious employees. This type of employment might not stigmatize their occupational status as much.

Job promotion, as the reward component, reflects satisfaction with the occupational position and income. No difference was found between the two groups on job promotion. However, in our detailed analysis of each statement, fixed-term workers were distressed more because they were less satisfied with the position and/or income compared to permanent workers. We focused on the workers in the middle stage of their career who might be concerned about their future position and salary to support their lifestyles and their families. Perception of the limited work prospect might influence hopelessness or discourage workers from continuous competition in the research occupation.

As frequently reported [29,31], job insecurity was a cause of distress among fixed-term workers more than among permanent workers also in our study. One of the items assessing job security was "My job security is poor." For this question, 251 (52%) fixed-term workers answered "distressed" while only 31 (14%) permanent workers provided the same answer. Even small numbers of permanent workers felt job insecurity. This might be because permanent workers may be also afraid of downsizing or restructuring. The other item assessing job insecurity inquired whether workers experienced or expect to experience an undesirable change in their work situation. Permanent workers were more likely to have experienced an undesirable change in one of the statements of the job security sub-category in ERI (permanent workers 44% and fixed-term workers 25%). This might be because permanent workers tended to work at the same institution for a longer period and realize when small changes occurred. One possible undesirable change could be replacing permanent workers with fixed-term workers.

High ERI and its association with subjective symptoms and obesity were suggested for fixed-term workers. It seems that a high ERI of fixed-term workers was more likely linked to physical or mental symptoms or obesity. Association with high ERI and poor health was concordant with previous studies [32] and we provided the evidence for that in particular employment status. Permanent workers were more obese in our analysis while higher ERI was associated with fixed-term workers' obesity. Fixed-term workers who have high job stress might resemble permanent workers regarding their health. In addition, obese fixed-term workers might experience physical weight gain from job stress, which might lower workers productivity and decrease the likelihood of promotion or finding a permanent position. Previous study suggested that obesity could be an obstacle to getting permanent work [10]. A similar tendency concerning obesity and subjective symptoms might be applicable to our study subjects.

Several limitations of our study should be noted. Since our study was a cross-sectional study, causal relationship cannot be established. However, this study at least describes the characteristics and sources of job stress among different types of employees. Second, this study was conducted in one institution in Japan. Generalizing our results to other organizations may be difficult and must be done with caution. The effort/reward ratios, 0.58 for permanent workers and 0.48 for fixed-term workers, were similar to a previous Japanese study conducted among men in which the ratio was 0.5 [30,33]. Thus, our study subjects were not an extreme population. Conducting the study in one institution could be advantageous as it can capture a population with workers of different employment status engaging in the same work and working in the same environment. Third, as our results for the esteem component showed that the targeted institution provided its employees with a supportive and fair environment. Even fixed-term workers obtained support from their colleagues, suggesting that inequity in the working environment might be small. This fair environment might underestimate the general environment of Japanese precarious workers. Although the targeted institution had a supportive environment, we found that workers actually felt job stress. It might be that personality characteristics influence perception of their working environment. In addition, the current study was conducted in a research institute where even precarious workers might easily control their workload. Workers in routine jobs reported that they could not control their work hours and tasks [34]. Examining other job categories might provide different results. Finally, we could not gather information on personal background, such as educational background, history of job career, and income. However, regardless of employment types, researchers attained at least graduate degrees, and clerical workers had at least some university level education. Thus, the educational attainment between permanent and fixed-term workers in our study may not be different. In spite of these limitations, our study evaluated workers' stress, particularly job stress, using ERI and described the association between a higher ERI and the health of permanent and fixed-term workers.

## Conclusions

The same proportion of permanent and fixed-term male workers was at high-risk for job stress as measured by the ERI questionnaire. However, we found that these two types of employees experience different sources of occupational stress. Permanent workers experienced a greater distress from overwork and job demand while fixed-term workers experienced greater distress from job insecurity. In addition, higher ERI was associated with existing subjective symptoms and being obese in fixed-term workers. Different patterns of job stress sources and associations with health should be recognized to understand the characteristics of workers with diverse employment status in the current society.

## Competing interests

The authors declare that they have no competing interests.

## Authors' contributions

MI performed statistical analysis and wrote the manuscript. ST and MN have been involved in drafting the manuscript. EY was the primary investigator of the study, contributed to study design, and assisted with writing of the manuscript. All authors read and approved the final manuscript.
